# Androgen deprivation therapy during and after post-prostatectomy radiotherapy in patients with prostate cancer: a case control study

**DOI:** 10.1186/s12885-018-4189-9

**Published:** 2018-03-09

**Authors:** Myong Kim, Cheryn Song, In Gab Jeong, Seung-Kwon Choi, Myungchan Park, Myungsun Shim, Young Seok Kim, Dalsan You, Jun Hyuk Hong, Choung-Soo Kim, Hanjong Ahn

**Affiliations:** 1 Department of Urology, Asan Medical Center, University of Ulsan College of Medicine, 88 Olympic-ro 43-gil, Songpa-gu, Seoul, 05505 Republic of Korea; 2 Department of Radiation Oncology, Asan Medical Center, University of Ulsan College of Medicine, Seoul, Republic of Korea

**Keywords:** Radiotherapy, Androgen-deprivation therapy, Prostate cancer, Survival, Prognostic factor

## Abstract

**Background:**

Here we assessed the influence of androgen deprivation therapy (ADT) during and/or after post-prostatectomy radiotherapy (RT) on biochemical recurrence (BCR) and radiographic progression in patients with prostate cancer.

**Methods:**

Patients with prostate cancer who underwent post-prostatectomy RT were analyzed. BCR and radiographic progression after RT were compared according to the concurrent or salvage ADT. Cox regression analyses were used to identify risk factors for BCR and radiographic progression.

**Results:**

Of the 227 patients who underwent post-prostatectomy RT, 95 (41.9%) received concurrent ADT for a median of 17.0 months. Despite more aggressive disease characteristics in the concurrent ADT group than in the RT-only group, the former had a better 5-year BCR-free survival rate than the latter (66.1 vs. 53.9%; *p* = 0.016), whereas the radiographic progression rate was not significantly different between two groups. On the other hand, salvage ADT after post-RT BCR significantly delayed radiographic progression (5-year radiographic progression-free survival; 75.2 vs. 44.5%; *p* = 0.002).

**Conclusions:**

Concurrent ADT improved BCR-free survival, and salvage ADT after post-RT BCR improved radiographic progression-free survival**.** To maximize the oncological benefit, ADT of sufficient duration should be implemented.

## Background

Despite the stage migration in prostate cancer noted in this prostate specific antigen (PSA) screening era, extraprostatic disease continues to occur in more than one-third of patients who undergo radical prostatectomy (RP) [1, 2]. Post-prostatectomy radiotherapy (RT) is advocated as a viable treatment option in both the adjuvant and salvage settings [3–5].

Three contemporary randomized controlled trials (RCTs) investigating adjuvant RT vs. observation after RP, namely the SWOG 8794 [6], EORTC 22911 [7], and ARO 96–02 [8], demonstrated that adjuvant RT reduced the risks of biochemical recurrence (BCR) and local relapse by approximately 20% at 5 years among patients with adverse pathologic features (i.e., seminal vesicle invasion, positive surgical margins with or without extraprostatic extension). The results of some large observational studies have indicated that salvage RT effectively controls locally recurrent disease after RP [9, 10].

However, patients with adverse pathologic characteristics or those who experience PSA recurrences after RP can harbor micrometastases that cannot be detected by imaging. In these cases, it may be beneficial to combine supplementary androgen deprivation therapy (ADT) with local RT, a notion being tested by several RCTs [11–15]. The final results from the RTOG 9601 showed that adding a 24 month anti-androgen (AA) treatment during salvage RT reduced mortality over a median follow-up of 12.6 years compared with salvage RT-only treatment (12-year overall survival [OS]: 76.3 vs. 71.3%; *p* = 0.04) [15] .

Long-term ADT can reduce quality of life and increase the risk of adverse events, including gynecomastia, cardiovascular accidents, fractures, and metabolic syndrome [15–19]. In this regard, the recent results from the GETUG-AFU 16 trial demonstrated that 6 months of luteinizing hormone releasing hormone (LHRH) agonist treatment during salvage RT significantly reduced clinical progression (5-year progression-free survival, 80.0 vs. 62.0%; *p* <  0.0001) [14]. These results are noteworthy as they support the survival benefit of concurrent ADT with post-prostatectomy RT.

However, previous RCT results did not conclude whether short-term ADT has an oncologic benefit equal to that of long-term ADT during post-prostatectomy RT [11–15]. Moreover, because the protocols of previous RCTs stated that salvage ADT should only be administered in cases of radiographic or pathologic evidence of metastatic disease [11, 12], they did not determine whether the androgen axis suppression that occurs by supplementary ADT can delay the next disease progression phase, such as radiographic progression after post-RT BCR. Here we assessed the oncological benefit of supplementary ADT during or after post-prostatectomy RT.

## Methods

### Patient selection

This study was approved by our institutional review board. The study population comprised 336 consecutive patients who underwent adjuvant or salvage RT following RP between August 1998 and March 2013. The exclusion criteria were the presence of other malignancies (*n* = 4, 1.2%), ineligibility according to American Society for Radiation Oncology (ASTRO)/American Urological Association (AUA) criteria for adjuvant or salvage RT [3] (*n* = 1, 0.3%), the administration of neoadjuvant ADT before RP (*n* = 7, 2.1%), failure to complete the planned RT dose (*n* = 2, 0.6%), and incomplete clinical data or loss to follow-up (*n* = 15, 4.5%). Patients whose PSA levels did not decline to undetectable levels (< 0.2 ng/mL) after RP (*n* = 80, 23.8%) were also excluded to ensure that the pure impact of supplementary ADT on the prognostic outcomes from post-prostatectomy RT was evaluated. Thus, 227 patients (67.6%) were included in the final analysis.

### Definitions and data acquisition

Adjuvant and salvage RT were defined according to the recent ASTRO/AUA criteria. Adjuvant RT was the administration of RT to RP patients who had adverse pathologic characteristics (pT2 with positive surgical margins, pT3, or pN1), prior to the PSA recurrence. Salvage RT was the administration of RT to patients with PSA recurrences after surgery without evidence of systemic disease [3].

Supplementary ADT was classified into concurrent and salvage ADT according to time of administration. Concurrent ADT was defined as ADT administered before, concurrent with, or after RT. Salvage ADT was defined as ADT administered after a post-RT BCR. The ADT regimens were manipulated according to PSA response. When castration resistance occurred, further treatments, including cytotoxic chemotherapy, were initiated, based on the physician’s decision.

Clinical variables during follow-up were retrieved from the patients’ medical records. The original [20] or revised [21] Gleason score criteria were applied according to the time of diagnosis. Tumor-lymph node-metastasis staging was determined using the revised American Joint Cancer Committee criteria [22].

### Statistical analyses

The concurrent ADT plus RT group and RT-only group were compared with respect to BCR-free survival from the date of RT. Radiographic progression-free survival was compared in the salvage and no salvage ADT groups; these groups comprised patients who experienced post-RT BCR (*n* = 81). Cox proportional hazards analyses were used to determine whether concurrent or salvage ADT affected BCR-free or radiographic progression-free survival. All tests were two-tailed with a significance level of < 0.05. The statistical analyses were performed using SPSS® software version 21.0 (IBM Corporation, Armonk, NY, USA).

## Results

### Patient characteristics

Of the 227 patients who underwent post-prostatectomy RT, 95 (41.9%) received concurrent ADT for a median 17.0 months (interquartile range [IQR], 12.5–22.0 months) (Table 1). Compared to the RT-only group, the concurrent ADT group had unfavorable clinical characteristics such as more frequent pN1 disease (12.6 vs. 3.0%) and higher pre-RT PSA level (0.72 vs. 0.39 ng/mL; Table 1). Of the 81 patients who experienced post-RT BCR, 50 patients (61.7%) received salvage ADT for a median 16.0 months (IQR, 3.8–51.3 months). The salvage ADT group was younger (61.0 vs. 65.0 years) and had a higher pre-RT PSA level (0.71 vs. 0.42 ng/mL) than the non-salvage ADT group. Other baseline characteristics did not differ between the two groups (Table 1).

**Table 1:** Comparisons of clinicopathologic characteristics of each sub-group categorized by the modes of supplementary androgen deprivation therapy during post-prostatectomy radiotherapy

	All patients (*n* = 227)			Patients with post-radiotherapy BCR (*n* = 81)		
	No concomitant ADT	Concurrent ADT	p-value^a^	No salvage ADT	Salvage ADT	*p*-value^a^
Number of patients	132	95	–	31	50	–
Patients characteristics						
Age (years)	64.0 (59.3–68.0)	64.0 (59.0–70.0)	0.500	65.0 (61.0–70.0)	61.0 (58.0–65.0)	0.012
Pre-operative PSA (ng/mL)	12.60 (7.00–22.90)	9.90 (6.90–18.90)	0.690	16.33 (6.40–28.00)	11.00 (6.55–25.20)	0.711
Pathology-related factors						
Gleason score	7 (7–9)	7 (7–9)	0.385	8 (7–9)	7 (7–9)	0.411
Pathologic T stage ≥3a	89 (67.4%)	56 (58.9%)	0.190	19 (61.3%)	36 (72.0%)	0.316
Pathologic N stage ≥1	4 (3.0%)	12 (12.6%)	0.005	2 (6.5%)	1 (2.0%)	0.302
Tumor volume (%)	10.0 (1.0–20.0)	9.0 (1.0–17.0)	0.504	9.0 (1.0–20.0)	2.0 (1.0–16.0)	0.110
Positive surgical margin	81 (61.4%)	59 (62.1%)	0.910	19 (61.3%)	27 (54.0%)	0.520
ADT-related factors						
Concomitant ADT duration (months)	–	17.0 (12.0–21.0)	–	–	–	–
Salvage ADT duration (months)	–	–	–	–	16.0 (3.8–51.3)	–
Initial regimen						
Complete androgen blockage	–	43 (45.3%)	–	–	8 (16.0%)	
LHRH agonist	–	31 (32.6%)		–	32 (64.0%)	
Antiandrogen	–	21 (22.1%)		–	10 (20.0%)	
Radiotherapy-related factors				–		
Pre-radiotherapy PSA (ng/mL)	0.39 (0.25–0.60)	0.72 (0.50–1.10)	< 0.001	0.42 (0.32–0.75)	0.71 (0.39–1.63)	0.007
Radiotherapy dose (Gy)	66.0 (66.0–70.0)	66.0 (66.0–66.0)	0.117	66.0 (66.0–70.0)	66.0 (66.0–70.0)	0.607

*ADT* androgen deprivation therapy, *BCR* biochemical recurrence, *PSA* prostate specific antigen, *LHRH* luteinizing hormone releasing hormone

All values are median (interquartile range) or the number (%)

^a^determined using the Mann-Whitney U test (continuous variables) or *χ*^2^ test (categorical variables)

### Effect of concurrent ADT on BCR

The median follow-up was 84.2 months (IQR, 59.3–108.9 months) from RP and 50.8 months (IQR, 36.3–66.8 months) from the post-prostatectomy RT. During follow-up, 81 patients (35.7%) experienced BCR and 38 (16.7%) showed radiographic progression. Of the patients with radiographic progression, 17 patients (7.5%) had local recurrence and 21 (9.3%) had distant metastases, respectively. The overall 5-year BCR-free and radiographic progression-free survival rates after post-prostatectomy RT were 59.0% and 84.0%, respectively.

**Fig. 1:**
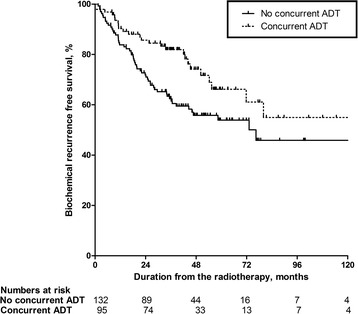
Comparison of the concurrent and no concurrent androgen deprivation therapy (ADT) groups with respect to biochemical recurrence (BCR)-free survival form the date of radiotherapy.The estimated 5-year BCR-free survival rates for the no concurrent and concurrent ADT groups were 53.9% and 66.1% (*p* = 0.016), respectively

The concurrent ADT group showed better 5-year BCR-free survival rate than the no concurrent ADT group (66.1 vs. 53.9%; *p* = 0.016; Fig. 1). Concurrent ADT (hazard ratio [HR] = 0.381; *p* = 0.034) was an independent prognostic factor for BCR after RT, along with pre-RT PSA level (≥1.0 ng/mL; HR = 4.383; *p* = 0.001; Table 2).

**Table 2:** Cox regression analysis of biochemical recurrence in patients treated with post-prostatectomy radiotherapy

	Univariate analysis		Multivariate analysis	
	HR (95% CI)	*p*-value	HR (95% CI)	*p*-value
Age (years)	0.974 (0.944–1.005)	0.099	0.978 (0.943–1.014)	0.228
BMI (kg/m^2^)	0.942 (0.871–1.018)	0.131		
Pre-operative PSA (ng/mL)				
< 20.00	(reference)		(reference)	
≥ 20.00	1.640 (1.032–2.608)	0.036	1.112 (0.625–1.976)	0.719
Pre-radiotherapy PSA (ng/mL)				
< 1.00	(reference)		(reference)	
≥ 1.00	2.122 (1.288–3.497)	0.003	4.383 (1.797–10.688)	0.001
Pathologic Gleason score				
≤ 7	(reference)			
≥ 8	1.393 (0.897–2.163)	0.140		
Pathologic T stage				
≤ pT2	(reference)			
≥ pT3	1.272 (0.798–2.029)	0.312		
Pathologic N stage				
pN0 or pNx	(reference)			
pN1	0.498 (0.157–1.579)	0.236		
Tumor volume (%)				
< 10.0	(reference)			
≥ 10.0	0.939 (0.600–1.469)	0.783		
Surgical margin tumor involvement				
Negative	(reference)			
Positive	0.815 (0.525–1.266)	0.363		
Radiation dose (Gy)				
< 66.0	(reference)			
≥ 66.0	0.770 (0.406–1.461)	0.424		
Testosterone nadir after RP (ng/mL)	1.088 (0.948–1.248)	0.229		
Duration of unrecovered testosterone level (months)	0.984 (0.970–0.998)	0.031	0.991 (0.971–1.011)	0.361
Concurrent ADT				
No	(reference)		(reference)	
Yes	0.564 (0.352–0.905)	0.018	0.381 (0.157–0.927)	0.034

*HR* hazard ratio, *CI* confidence interval, *BMI* body mass index, *PSA* prostate specific antigen, *RP* radical prostatectomy, *ADT* androgen deprivation therapy

### Effect of salvage ADT on radiographic progression

**Fig. 2:**
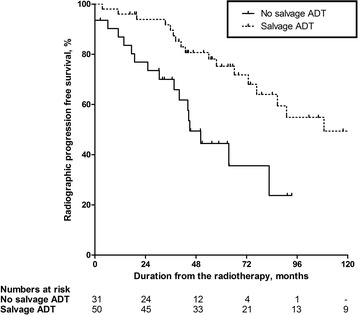
Comparison of the salvage and no salvage androgen deprivation therapy (ADT) groups with respect to radiographic progression-free survival from the date of radiotherapy (81 patients experienced BCR after radiotherapy). The estimated 5-year radiographic progression-free survival rates for the no salvage and salvage ADT groups were 44.5% and 75.2% (*p* = 0.002), respectively

A total of 81 patients experienced post-RT BCR, and the salvage ADT group showed better 5-year radiographic progression-free survival than the no salvage ADT group (75.2 vs. 44.5%; *p* = 0.002; Fig. 2). The multivariate analysis demonstrated that salvage ADT (HR = 0.306; p = 0.001) was an independent prognostic factor for radiographic progression, along with the pN stage (pN1; HR = 16.457; p = 0.001), and the tumor volume (≥10.0%; HR = 4.137; *p* <  0.001; Table 3). However, previous administrations of concurrent ADT did not affect radiographic progression (univariate analysis; *p* = 0.725; Table 3).

**Table 3:** Cox regression analysis of radiographic progression in patients treated with post-prostatectomy radiotherapy (*n* = 81) who experienced biochemical recurrence after radiotherapy

	Univariate analysis		Multivariate analysis	
	HR (95% CI)	*p*-value	HR (95% CI)	*p*-value
Age (years)	1.035 (0.978–1.096)	0.231		
BMI (kg/m^2^)	0.913 (0.773–1.078)	0.282		
Pre-operative PSA (ng/mL)				
< 20.00	(reference)			
≥ 20.00	1.111 (0.551–2.241)	0.768		
Pre-radiotherapy PSA (ng/mL)				
< 1.00	(reference)			
≥ 1.00	0.906 (0.427–1.923)	0.796		
Pathologic Gleason score				
≤ 7	(reference)		(reference)	
≥ 8	2.438 (1.169–5.084)	0.017	1.288 (0.636–2.609)	0.482
Pathologic T stage				
≤ pT2	(reference)			
≥ pT3	1.262 (0.599–2.659)	0.540		
Pathologic N stage				
pN0, or pNx	(reference)		(reference)	
pN1	6.096 (1.316–28.234)	0.021	16.457 (3.358–80.652)	0.001
Tumor volume (%)				
< 10.0	(reference)		(reference)	
≥ 10.0	3.888 (1.923–7.862)	< 0.001	4.137 (1.999–8.562)	< 0.001
Surgical margin tumor involvement				
Negative	(reference)			
Positive	1.678 (0.819–3.437)	0.157		
Radiation dose (Gy)				
< 66.0	(reference)			
≥ 66.0	1.564 (0.619–3.951)	0.344		
Testosterone nadir after RP (ng/mL)	1.170 (0.834–1.643)	0.363		
Duration of unrecovered testosterone level (months)	1.002 (0.989–1.015)	0.802		
Concurrent ADT				
No	(reference)			
Yes	1.134 (0.563–2.287)	0.725		
Salvage ADT				
No	(reference)		(reference)	
Yes	0.344 (0.171–0.692)	0.003	0.306 (0.150–0.627)	0.001

*HR* hazard ratio, *CI* confidence interval, *BMI* body mass index, *PSA* prostate specific antigen, *RP* radical prostatectomy, *ADT* androgen deprivation therapy

## Discussion

### Concurrent ADT with post-prostatectomy RT

Previous RCTs such as the RTOG 9601 [15] and GETUG-AFU 16 [14] reported that compared with salvage RT-only, long-term (24 months [15]) or short-term (6 months [14]) ADT with salvage RT significantly improved BCR. In this study, we also confirmed the benefit of current ADT in terms of BCR-free survival. However, there have been limited data on the proper duration of concurrent ADT during post-prostatectomy RT. Short-term (< 12 months) concurrent ADT was reportedly associated with increases in BCR (HR = 2.27; *p* = 0.003) and distant metastasis (HR = 2.48; *p* = 0.03) compared with longer-term (≥12 months) ADT [23]. With respect to ADT duration, we found that patients who underwent < 12 months of concurrent ADT showed poorer 5-year BCR-free survival than patients who underwent longer-term (≥12 months) ADT, although the difference failed to reach statistical significance (*p* = 0.232; Appendix). These findings concur with the results of a previous study [23]. These findings suggest that the concurrent ADT duration should be extended to 12 months or longer.

### Role of salvage ADT

The RTOG 9601 study protocol stated that salvage ADT should only be administered when there is radiographic or pathologic evidence of metastatic disease [11]. The administration of salvage ADT was not restricted in this way in our study; consequently, a substantial proportion of the patients who developed post-RT BCR (61.7%) were administered salvage ADT. Clearly, the strict specifications of the RTOG 9601 were necessary to determine the pure effects of concurrent ADT in a post-prostatectomy RT setting; however, our study resembles real-life practice more closely.

In real clinical practice, ADT is not only delivered concurrently with RT. Indeed, when post-RT BCR occurs, salvage ADT may be considered a viable treatment option in patients with hormone-naïve or hormone-sensitive prostate cancer [4, 5]. The oncological role of salvage ADT after post-RT BCR remains unclear. Given that there are numerous instances of salvage ADT in clinical settings, it is also important to determine whether salvage ADT can benefit patients with post-RT BCR. Our results demonstrated that salvage ADT independently improved radiographic progression (HR = 0.306; *p* = 0.001; Table 3). Previous administrations of concurrent ADT did not affect radiographic progression (univariate analysis: *p* = 0.725; Table 3). These findings strongly imply that the differences in the radiographic progression in the salvage ADT group were also caused by the direct suppression of the androgen axis by the salvage ADT itself. Hence, we suggest that salvage ADT can be a viable treatment option that may alter radiographic progression when BCR occurs after post-prostatectomy RT in patients with hormone-naïve or hormone-sensitive prostate cancer.

### Limitations of the current study

Our study was limited by its retrospective nature and the relatively small number of patients included. In addition, information for some of the variables was absent, because some of the patients’ medical records were incomplete. Moreover, the effects of the different types of ADT applied to the study cohort on BCR and radiographic progression were not considered. This reflects the fact that, in most patients, the ADT regimen was manipulated based on the PSA levels, which resulted in substantial regimen heterogeneity that precluded closer analyses.

## Conclusions

Concurrent ADT during post-prostatectomy RT significantly improved BCR-free survival, and salvage ADT after post-RT BCR improved radiographic progression-free survival. Therefore, to maximize the oncological benefit, ADT of sufficient durations should be implemented, and salvage ADT should be considered as a viable treatment option after post-RT BCR. The results from ongoing RCTs are needed to confirm our results.

## Figures and Tables

**Fig. 3:**
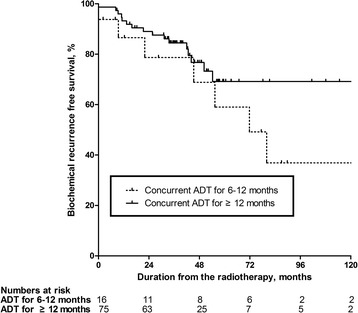
Comparison of biochemical recurrence (BCR)-free survival form radiotherapy date with respect to duration of concurrent androgen deprivation therapy (ADT). Patients with < 12 months of concurrent ADT showed poorer 5-year BCR-free survival rates than those with longer-term (≥12 months) ADT, although the difference failed to reach statistical significance (*p* = 0.232)

## Abbreviations

AA: Anti-androgen
ADT: Androgen deprivation therapy
ASTRO: American Society for Radiation Oncology
AUA: American Urological Association
BCR: Biochemical recurrence
HR: Hazard ratio
IQR: Interquartile range
LHRH: Luteinizing hormone releasing hormone
OS: Overall survival
PSA: Prostate specific antigen
RCT: Randomized controlled trial
RP: Radical prostatectomy
RT: Radiotherapy
